# Early serum TIMP-2 as a biomarker for 90-day chronic kidney disease after percutaneous coronary intervention in ST-elevation myocardial infarction: a prospective cohort study

**DOI:** 10.1186/s12872-026-05904-8

**Published:** 2026-05-06

**Authors:** Miras Mugazov, Eldar Saparov, Dmitriy Vassilyev, Alina Ogizbayeva, Natalya Vassilyeva, Dinara Omertayeva

**Affiliations:** 1 Department of Emergency Medical Care, Anesthesiology and Resuscitation, NJSC Karaganda Medical University, Karaganda, Kazakhstan; 2 Department of Obstetrics, Gynecology and Perinatology, NJSC Karaganda Medical University, Karaganda, Kazakhstan

**Keywords:** TIMP-2, Contrast-induced acute kidney injury, ST-elevation myocardial infarction, Percutaneous coronary intervention, Chronic kidney disease, Tubular stress biomarkers, Cardio–renal syndrome

## Abstract

**Background:**

Contrast-induced acute kidney injury (CI-AKI) remains a concern in patients with ST-elevation myocardial infarction (STEMI) undergoing emergent percutaneous coronary intervention (PCI), but creatinine-based CI-AKI definitions incompletely capture longer-term renal trajectories. Early tubular stress biomarkers such as tissue inhibitor of metalloproteinases-2 (TIMP-2) may provide incremental risk information beyond conventional functional markers.

**Methods:**

In this prospective single-centre cohort, we enrolled 88 consecutive STEMI patients undergoing urgent coronary angiography with or without PCI. Routine laboratory parameters were obtained at admission and 48 h. Serum TIMP-2 was measured 2 h after the procedure using a standardized sandwich ELISA. Early CI-AKI was defined by conventional creatinine-based criteria within 48 h, whereas 90-day chronic kidney disease (CKD) was defined as an estimated glomerular filtration rate (eGFR) < 60 mL/min/1.73 m² or persistent creatinine elevation at 90 days. We evaluated paired changes, between-group differences by 90-day CKD status, correlations, multivariable linear and logistic regression, and receiver operating characteristic (ROC) discrimination.

**Results:**

Early CI-AKI occurred in 6/88 patients (6.8%), whereas 90-day CKD was diagnosed in 15/88 (17%), indicating substantial delayed renal dysfunction beyond the 48-h window. The median 2-h serum TIMP-2 concentration was 1.20 ± 0.38 ng/mL. TIMP-2 showed modest correlations with creatinine and urea and remained independently associated with higher 90-day creatinine in multivariable linear regression (B = + 12.0 µmol/L per 1-ng/mL increase; *p* = 0.016) after adjustment for baseline creatinine and clinical covariates. In parsimonious logistic models, TIMP-2 demonstrated a borderline association with 90-day CKD (odds ratio ≈ 6.38; *p* = 0.056), while older age was a significant predictor. Standalone ROC discrimination for 90-day CKD was limited (AUC 0.528; 95% confidence interval 0.39–0.65; *p* = 0.668).

**Conclusions:**

In STEMI patients undergoing emergent angiography/PCI, 90-day CKD was considerably more frequent than early creatinine-defined CI-AKI, underscoring the need for extended renal follow-up. A single 2-hour serum TIMP-2 measurement reflects early tubular stress and independently associates with 90-day creatinine, but offers only modest standalone discrimination for 90-day CKD. TIMP-2 is therefore more likely to have a role within multivariable or biomarker panel-based risk stratification pathways rather than as an isolated classifier.

## Introduction

Despite significant enhancements in peri-procedural care over the last two decades-including the almost universal adoption of intravenous volume expansion, the widespread switch to iso- or low-osmolar non-ionic contrast agents, and the preferential use of radial access-longer-term deterioration of renal function remains surprisingly common following primary percutaneous coronary intervention (PCI) for ST-elevation myocardial infarction (STEMI) [1, 2]. Contemporary registries have consistently reported that 10–25% of patients experience a sustained decline in estimated glomerular filtration rate (eGFR) or new-onset chronic kidney disease (CKD) within 1 year of the index event, even in the absence of conventional short-term definitions of contrast-induced acute kidney injury (CI-AKI) [3–6]. These observations have profoundly challenged the longstanding paradigm that iodinated contrast media represent the dominant or sole driver of post-PCI AKI [7–9]. Large-scale observational studies and rigorously conducted propensity-matched analyses have repeatedly demonstrated that, following meticulous adjustment for baseline renal function, haemodynamic status, and inflammatory burden, intravenous contrast administration is no longer an independent predictor of AKI or dialysis in the majority of contemporary cohorts [10–13]. Rather, the dominant risk factors appear to be acute heart failure (Killip class ≥ 3), profound systemic inflammation, neurohormonal activation, and pre-existing impairment of renal reserve-factors that are intrinsic to the pathophysiology of STEMI itself rather than to the contrast load per se [14–16].

This paradigm shift has critical implications. First, it implies that many cases previously labelled as “contrast-induced” AKI may in fact represent acute cardio-renal syndrome type 1 or acute kidney injury triggered by the myocardial infarction and reperfusion process itself. Second, and more importantly for clinical practice, it highlights the inadequacy of traditional creatinine-based endpoints that focus exclusively on the first 48–72 h after contrast exposure. Serum creatinine is a late and relatively insensitive marker of glomerular filtration, influenced by muscle mass, volume of distribution, tubular secretion, and non-renal clearance pathways [17, 18]. In the setting of acute myocardial infarction, haemodilution from aggressive hydration, changes in creatinine generation due to reduced muscle perfusion, and alterations in tubular handling further confound interpretation [19, 20]. Consequently, a substantial proportion of patients who do not meet conventional CI-AKI criteria nevertheless progress to clinically meaningful chronic kidney disease over weeks to months—an outcome that carries independent prognostic weight for cardiovascular events, heart-failure hospitalisation, and all-cause mortality [21–23].

Limitations in creatinine-centric definitions have catalysed an intensive search for biomarkers that detect renal tubular stress or injury before detectable changes in filtration occur. Among the most promising candidates are proteins involved in the G1 cell-cycle arrest response of renal tubular epithelium. Tissue inhibitor of metalloproteinases-2 (TIMP-2) and insulin-like growth factor-binding protein 7 (IGFBP7) are secreted in direct response to a variety of cellular stresses (oxidative damage, DNA injury, ischaemia–reperfusion, inflammation) and induce reversible cell-cycle arrest, thereby protecting tubular cells from apoptosis and facilitating repair [24, 25]. Their product ([TIMP-2]·[IGFBP7]), marketed as NephroCheck^®^, received FDA approval in 2014 and European CE marking for early AKI risk stratification in critically ill patients [26, 27]. Multiple large multicentre validation studies in cardiac surgery, sepsis, and general ICU populations have demonstrated excellent discrimination (AUC typically 0.80–0.95) for moderate-to-severe AKI within 12–24 h of biomarker measurement, far outperforming clinical risk scores alone [28–32].

Evidence remains remarkably sparse and contradictory in the specific context of primary PCI. A few single-centre studies have reported increased urinary [TIMP-2]·[IGFBP7] levels in the early phase following contrast exposure, but predictive performance regarding conventional CI-AKI has been inconsistent, with AUC values ranging from 0.60 to 0.85 [33–36]. Furthermore, virtually all studies conducted to date have examined only short-term (≤ 7-day) creatinine-based endpoints, meaning the relationship between early cell-cycle arrest signals and longer-term renal trajectories has not been explored at all in this population.

We thus designed the present study with two explicit objectives:

- This was done to quantify the true incidence of 90-day CKD (new-onset or progression) after contemporary primary percutaneous coronary intervention for STEMI in a real-world cohort by using rigorous, guideline-concordant definitions extending beyond the conventional 48–72-hour window.
- To test the hypothesis that a single very early (2 h post-procedural) measurement of serum TIMP-2—timed to capture the synergistic tubular stress induced by acute myocardial ischaemia–reperfusion, systemic inflammation, and contrast exposure - provides independent and incremental prognostic information for both short-term CI-AKI and, more importantly, longer-term CKD.

Addressing these gaps would position us to contribute meaningfully to the evolving understanding of cardio-renal interactions in acute coronary syndromes and to lay the foundation for future biomarker-guided nephroprotective strategies in this high-risk population.

## Materials and methods

### Study design and population

We conducted a prospective, single-centre observational cohort study of consecutive adult patients presenting with ST-elevation myocardial infarction (STEMI) and undergoing urgent coronary angiography with or without percutaneous coronary intervention (PCI) at the Department of Cardiology, Multiprofile Hospital No. 2, Karaganda. The study was designed to reflect routine clinical practice in an emergent STEMI setting and to capture early post-procedural tubular stress as well as longer-term renal outcomes.

Eligible patients were aged ≥ 18 years and had a diagnosis of STEMI established according to contemporary ESC/ACC guidelines based on typical symptoms, electrocardiographic ST-segment elevation criteria, and elevated cardiac biomarkers. All patients underwent emergent coronary angiography during the index hospitalization with the intention to perform primary PCI when anatomically feasible. We excluded patients with dialysis-dependent chronic kidney disease (CKD), known baseline estimated glomerular filtration rate (eGFR) < 30 mL/min/1.73 m², pregnancy or lactation, and those who refused or were unable to provide informed consent.

### Ethical considerations

All procedures performed in the study was conducted in accordance with the guidelines outlined in the Helsinki Declaration and its amendments. This study was approved by the Bioethics Committee of the NJSC “Karaganda Medical University” (Protocol No.19 date: 11.11.2024, with the assigned number No.86). Informed consent was obtained from all participants included in the study.

The present analysis focuses on the association between a single early serum tissue inhibitor of metalloproteinases-2 (TIMP-2) measurement and renal outcomes up to 90 days after the index procedure.

### Clinical management and PCI procedures

Patients were managed according to contemporary STEMI guidelines, including antiplatelet and antithrombotic therapy, beta-blockers, statins, and other evidence-based treatments as clinically indicated. The choice of vascular access (radial vs. femoral), guiding catheters, stent type, and use of adjunctive devices (e.g., thrombus aspiration, mechanical circulatory support) was left to the discretion of the treating interventional cardiologist.

During coronary angiography and PCI, the culprit vessel, number of diseased vessels, lesion characteristics, and procedural success were recorded. Procedural metrics included total contrast volume (mL) and radiation dose (mGy), which are reported as indicators of procedural complexity. Periprocedural hydration and nephroprotective measures followed institutional standards and were not protocolised for this study, reflecting real-world practice.

#### Contrast media

All patients received a nonionic, low-osmolar monomeric iodinated contrast agent with an iodine concentration of 300–370 mg I/mL (e.g., ioversol/iopromide class). These agents typically exhibit an osmolality of approximately 600–800 mOsm/kg H₂O and lower viscosity than iso-osmolar dimers, aiming to balance adequate image quality with a favourable nephrotoxicity profile. Contrast was administered intra-arterially through standard catheters, and total contrast volume per procedure was recorded for each patient and subsequently considered as a covariate in the statistical analyses.

#### Laboratory measurements and echocardiography

Routine laboratory testing was performed at hospital admission (pre- or immediately post-angiography) and at 48 h after the index procedure in the hospital’s central laboratory, using certified automated analysers with regular calibration and internal quality control according to manufacturer and institutional standards.

The laboratory panel included complete blood count (haemoglobin, haematocrit, white blood cells [WBC], platelets), serum biochemistry (creatinine, urea, electrolytes, liver enzymes, glucose, lipids), coagulation tests, and high-sensitivity cardiac troponin. Urinalysis was performed using automated dipstick and microscopic sediment examination, recording proteinuria and urinary leukocyturia (WBC in urine). eGFR was calculated from serum creatinine using the CKD-EPI equation and expressed in mL/min/1.73 m².

Transthoracic echocardiography was performed according to standard clinical practice during the index hospitalization, with left ventricular ejection fraction (LVEF) and left ventricular end-diastolic volume (LV-EDV) recorded where available, providing an assessment of cardiac function and filling status.

#### TIMP-2 sampling and assay

Venous blood for serum TIMP-2 analysis was obtained 2 h after completion of coronary angiography/PCI. The sampling time was chosen to capture early tubular stress in the immediate post-contrast and post-ischemia–reperfusion period. Blood samples were drawn into standard serum tubes, allowed to clot, and processed within 60 min. After centrifugation, serum was aliquoted into prelabelled cryovials and stored at ≤ − 20 °C in a dedicated biobank facility until batch analysis.

To minimise inter-assay variability, all stored samples were thawed once and processed in a single analytical run using an automated ELISA processor. TIMP-2 concentrations were quantified using a commercially available standardized sandwich ELISA (Quantikine^®^ Human TIMP-2, Cat. DTM200, R&D Systems, Minneapolis, MN, USA). Samples were diluted 1:50 according to the manufacturer’s instructions. A four-parameter logistic calibration curve was constructed from kit-provided standards, and optical density was read at 450 nm with 540/570-nm wavelength correction as recommended. Concentrations were expressed in ng/mL. Internal controls and calibration verification were performed in accordance with laboratory quality assurance procedures.

We examined both early and delayed renal outcomes. Early contrast-induced acute kidney injury (CI-AKI) within 48 h was defined using conventional creatinine-based criteria as either a ≥ 25% increase from baseline or an absolute increase of ≥ 44 µmol/L in serum creatinine, in line with guideline-based definitions. The primary long-term renal outcome was 90-day CKD status. Patients were classified as having 90-day CKD if, at approximately 90 days after the index procedure, they had an eGFR < 60 mL/min/1.73 m² or a persistent elevation in serum creatinine compared with their baseline value, based on outpatient laboratory results or planned follow-up visits. When in-person follow-up was not feasible, telephone contact was used to obtain information about interim clinical events and external laboratory testing, where available. Secondary renal outcomes included continuous 90-day serum creatinine and eGFR values. We also characterised the incidence of early CI-AKI and explored its relationship with 90-day CKD.

#### Sample size considerations

The study was conceived as an exploratory observational cohort in a single centre with an emphasis on feasibility and hypothesis generation regarding the role of early TIMP-2 in predicting 90-day renal outcomes. No formal a priori sample size calculation for biomarker discrimination was performed. Instead, the target was to enrol consecutive eligible STEMI patients over a defined time frame, resulting in a final sample of 88 patients. Given the observed 90-day CKD event rate of 17% (15/88), we adopted a parsimonious approach to multivariable modelling to reduce the risk of overfitting.

### Statistical methods

All statistical analyses were performed using standard statistical software (e.g., SPSS version 22, Medculc). Continuous variables are presented as.

mean ± standard deviation (SD) for approximately normally distributed variables and as median with interquartile range (IQR) for skewed distributions, as assessed by the Shapiro–Wilk test. Categorical variables are presented as counts and percentages.

Paired admission-to-48 h changes in laboratory parameters were assessed using paired t-tests for normally distributed data or Wilcoxon signed-rank tests otherwise. Between-group comparisons according to 90-day CKD status were performed using independent-samples t-tests or Mann–Whitney U tests for continuous variables, and chi-square or Fisher’s exact tests for categorical variables, as appropriate. Correlations between 2-h TIMP-2 levels and renal (serum creatinine, eGFR, urea) or inflammatory indices (e.g., WBC count) were evaluated using Pearson’s or Spearman’s correlation coefficients, depending on distributional assumptions. The strength and direction of correlations were interpreted in light of biological plausibility.

To examine the independent association of early TIMP-2 with longer-term renal outcomes, we constructed multivariable linear regression models with 90-day serum creatinine as the dependent variable. Prespecified covariates included baseline creatinine as the main functional marker of pre-existing kidney function, age, sex, total contrast volume, and 2-h TIMP-2. Regression coefficients (B) with 95% confidence intervals (CIs) and p-values were reported. Residual plots and variance inflation factors were inspected to assess assumptions of linearity, homoscedasticity, and absence of problematic multicollinearity.

To evaluate the association of TIMP-2 with the binary 90-day CKD outcome, we fitted parsimonious logistic regression models, given the limited number of events (*n* = 15). TIMP-2 and clinically relevant covariates (e.g., age, baseline creatinine, and contrast volume) were sequentially considered, with attention to an approximate events-per-variable ratio to mitigate overfitting. Odds ratios (ORs) with 95% CIs and p-values were reported.

Discrimination of 2-h TIMP-2 for 90-day CKD was assessed using receiver operating characteristic (ROC) curve analysis, with calculation of the area under the curve (AUC), standard errors, and 95% CIs. An AUC of 0.5 was interpreted as no discrimination, whereas higher values indicated increasing discriminative ability. A two-sided p-value < 0.05 was considered statistically significant for all analyses.

## Results

### Baseline characteristics and procedural data

A total of 88 patients with STEMI undergoing emergent coronary angiography with or without PCI were enrolled. The mean age was 63 ± 12 years, and 68% were male. Baseline clinical and demographic characteristics are summarised in Table 1. The cohort had a high prevalence of traditional cardiovascular risk factors: 62 patients (70%) had arterial hypertension and 24 (27%) had diabetes mellitus. The mean body mass index (BMI) was 28.4 ± 5.1 kg/m², reflecting an overweight population. Signs of haemodynamic compromise at presentation were not uncommon; 18 patients (20%) had Killip class ≥ 2.

**Table 1:** Baseline characteristics (*n* = 88)

Parameter	Value (Mean ± SD, Median [IQR], or *n*, %)
Age (years)	63 ± 12
Female sex	28 (32%)
BMI (kg/m²)	28.4 ± 5.1
Diabetes mellitus	24 (27%)
Arterial hypertension	62 (70%)
Killip class ≥ 2	18 (20%)
Baseline creatinine (µmol/L)	98 (82–118)
Baseline eGFR (mL/min/1.73 m²)	72 (58–86)
LVEF (%)	48 ± 11
LVEF < 40%	19 (22%)
Peak hsTnI (ng/mL)	8.4 (3.2–24.6)
Cardiogenic shock	8 (9%)
Transfusions during hospitalization	12 (14%)
Nephrotoxic drug exposure	
NSAIDs	8 (9%)
Aminoglycosides	2 (2%)
Kidney disease modifying drugs	
SGLT2 inhibitors	8 (9%)
MRA	15 (17%)

Baseline renal function was moderately reduced on average, with a median serum creatinine of 98 µmol/L (IQR 82–118) and median eGFR of 72 mL/min/1.73 m² (IQR 58–86), suggesting that a considerable proportion of patients presented with reduced renal function at admission, although it should be noted that in the acute STEMI setting, admission creatinine may be influenced by hemodynamic instability and does not necessarily reflect pre-existing chronic kidney disease. Procedural characteristics reflected contemporary emergent PCI practice. The mean contrast dose was 162 ± 58 mL, and the mean radiation dose was 1450 ± 720 mGy, suggesting a mix of relatively straightforward and more complex interventions. Overall, the study population represents a typical real-world STEMI PCI cohort at risk for contrast-associated renal events.

### Early CI-AKI and 90-day CKD incidence

**Fig. 1:**
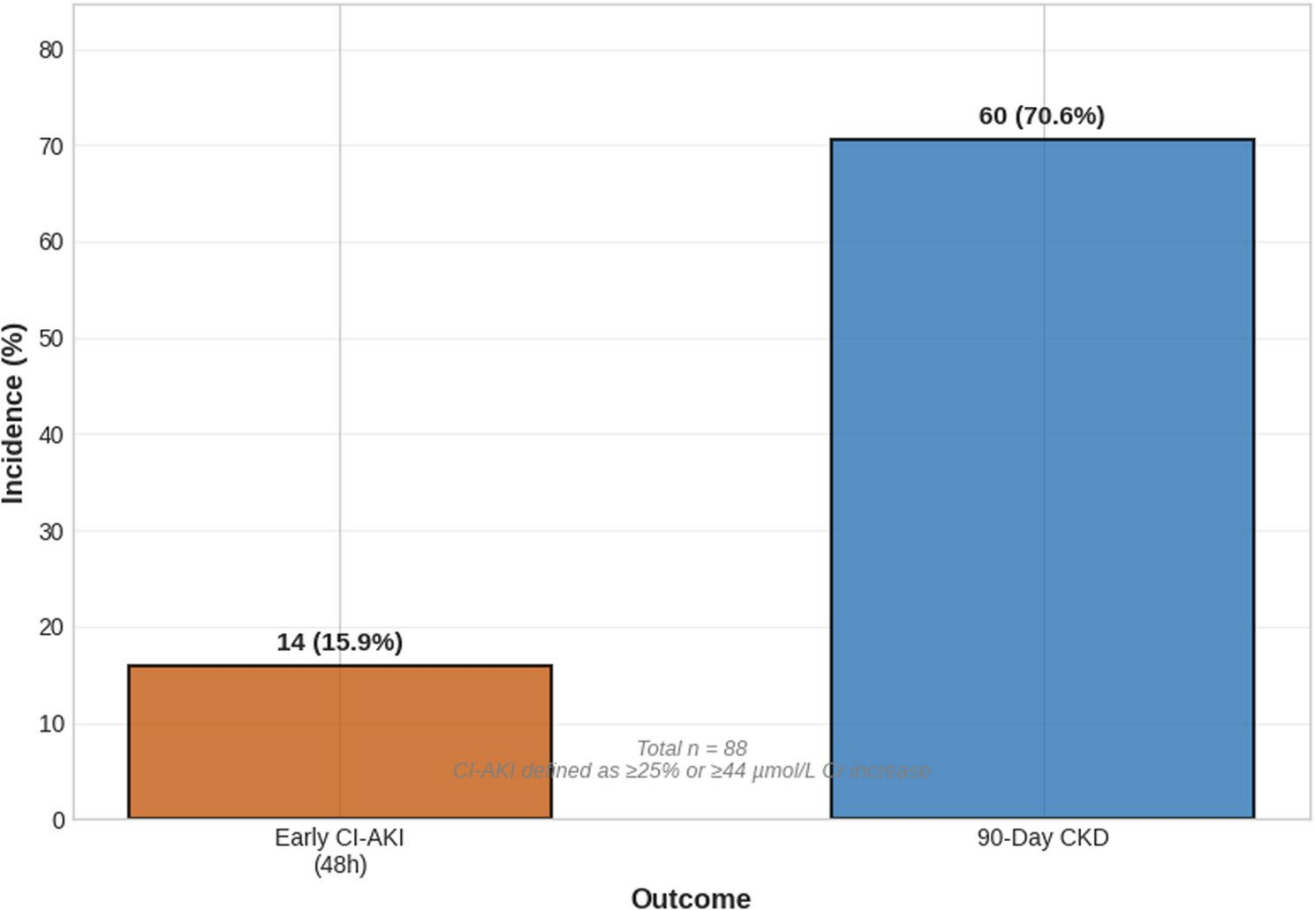
Incidence of early creatinine-defined contrast-induced acute kidney injury (CI-AKI) and 90-day chronic kidney disease (CKD) in the study cohort

Within the first 48 h after the index procedure, early CI-AKI as defined by conventional creatinine-based criteria occurred in 6 out of 88 patients (6.8%). In contrast, 90-day CKD, defined as eGFR < 60 mL/min/1.73 m² or persistent creatinine elevation at 90 days, was diagnosed in 15 of 88 patients (17%). Thus, longer-term renal dysfunction was approximately 2.5 times more frequent than early CI-AKI, underscoring a significant burden of delayed kidney impairment that is not captured by short-term monitoring alone. The incidence of early CI-AKI and 90-day CKD is summarised in Fig. 1.

### Comparison of baseline and 48-hour laboratory parameters

Laboratory parameters at baseline and at 48 h are detailed in Table 2. On average, median haemoglobin decreased from 140 g/L (IQR 128–152) at admission to 126 g/L (IQR 115–140) at 48 h, reflecting haemodilution, peri-procedural blood loss, or phlebotomy; this change was statistically significant (Wilcoxon Z = − 6.952; *p* < 0.001). Platelet counts declined from 238 ± 72 × 10⁹/L to 210 ± 65 × 10⁹/L (Z = − 4.166; *p* < 0.001), and WBC counts decreased from 11.5 ± 4.2 × 10⁹/L to 8.9 ± 3.1 × 10⁹/L (Z = − 3.951; *p* < 0.001), consistent with the expected evolution of the acute inflammatory response after STEMI and reperfusion.

**Table 2:** Laboratory parameters at baseline and 48 hours

Parameter	Baseline	48 h
Hemoglobin (g/L)	140 (128–152)	126 (115–140)
Platelets (×10⁹/L)	238 ± 72	210 ± 65
WBC (×10⁹/L)	11.5 ± 4.2	8.9 ± 3.1
Creatinine (µmol/L)	98 (82–118)	102 (84–124)
eGFR (mL/min/1.73 m²)	72 (58–86)	66 (52–82)
Urea (mmol/L)	0.0 (5.2–9.1)	0.4 (5.5–9.6)
Sodium (Na⁺, mmol/L)*	-	-
2-hour serum TIMP-2 (ng/mL)	1.20 (0.85–1.55)	-

Serum creatinine showed a modest average increase from a median of 98 µmol/L (IQR 82–118) at baseline to 102 µmol/L (IQR 84–124) at 48 h, while median eGFR decreased from 72 (IQR 58–86) to 66 (IQR 52–82) mL/min/1.73 m². Although these average changes were modest, interindividual variability was considerable, with some patients meeting CI-AKI criteria and others demonstrating stable or improved renal function. Serum urea exhibited a small but significant increase over 48 h (Z = + 2.025; *p* = 0.043). Overall, these dynamic changes reflect the combined effects of acute illness, revascularisation, haemodynamic shifts, and supportive therapy.

Laboratory рaired changes (Table 3) showed significant declines in hemoglobin, platelets, WBC, and electrolytes (*p* < 0.001 for most).

**Table 3:** Paired admission-to-48-hour changes (Wilcoxon signed-rank tests)

Parameter	Z statistic	*p*-value
Hemoglobin	-6.952	< 0.001
Platelets	-4.166	< 0.001
WBC	-3.951	< 0.001
Sodium (Na⁺)	-4.934	< 0.001
Urea	+ 2.025	0.043

### Early TIMP-2 levels

The mean 2-hour serum TIMP-2 concentration for the entire cohort was 1.20 ± 0.38 ng/mL. TIMP-2 levels showed a reasonably wide distribution, indicating heterogeneity in the degree of early tubular stress across patients. Although formal subgroup comparisons of TIMP-2 by 90-day CKD status were limited by sample size, patients who later developed 90-day CKD tended to exhibit numerically higher TIMP-2 values than those who remained free of CKD, in line with the observed associations in regression models.

### Group differences according to 90-day CKD

Between-group comparisons by 90-day CKD status are summarised in Table 4. Patients who developed 90-day CKD had lower baseline eGFR (*p* = 0.044), consistent with more impaired renal reserve at study entry. At 48 h, they also had lower eGFR and higher serum creatinine compared with patients without CKD, reflecting less favourable short-term renal trajectories.

The median 2-hour serum TIMP-2 concentration for the entire cohort was 1.20 ng/mL (IQR 0.85–1.55). TIMP-2 levels showed a reasonably wide distribution, indicating heterogeneity in the degree of early tubular stress across patients. Although formal subgroup comparisons of TIMP-2 by 90-day CKD status were limited by sample size, patients who later developed 90-day CKD tended to exhibit numerically higher TIMP-2 values than those who remained free of CKD, in line with the observed associations in regression models.

**Table 4:** Between-group differences according to 90-day CKD status

Variable	Statistical test	*p*-value	Direction of difference
Hemoglobin at 48 h	Mann–Whitney U	0.007	Higher in no-CKD group
Proteinuria	Mann–Whitney U	0.037	Higher in CKD group
Urine WBC	Mann–Whitney U	0.015	Higher in CKD group
Baseline eGFR	Mann–Whitney U	0.044	Lower in CKD group
Total contrast volume	Mann–Whitney U	0.046	Higher in CKD group

In addition, the 90-day CKD group received higher total contrast volumes (*p* = 0.046), suggesting an interaction between baseline renal vulnerability and procedural complexity or contrast burden. Proteinuria and urinary WBC counts were more frequent or more pronounced in the CKD group (proteinuria *p* = 0.037; urine WBC *p* = 0.015), supporting the notion of more advanced or persistent renal involvement with both glomerular and tubular/inflammatory components. Haemoglobin at 48 h was higher in the no-CKD group (*p* = 0.007), possibly reflecting less haemodilution, bleeding, or chronic anaemia.

**Fig. 2:**
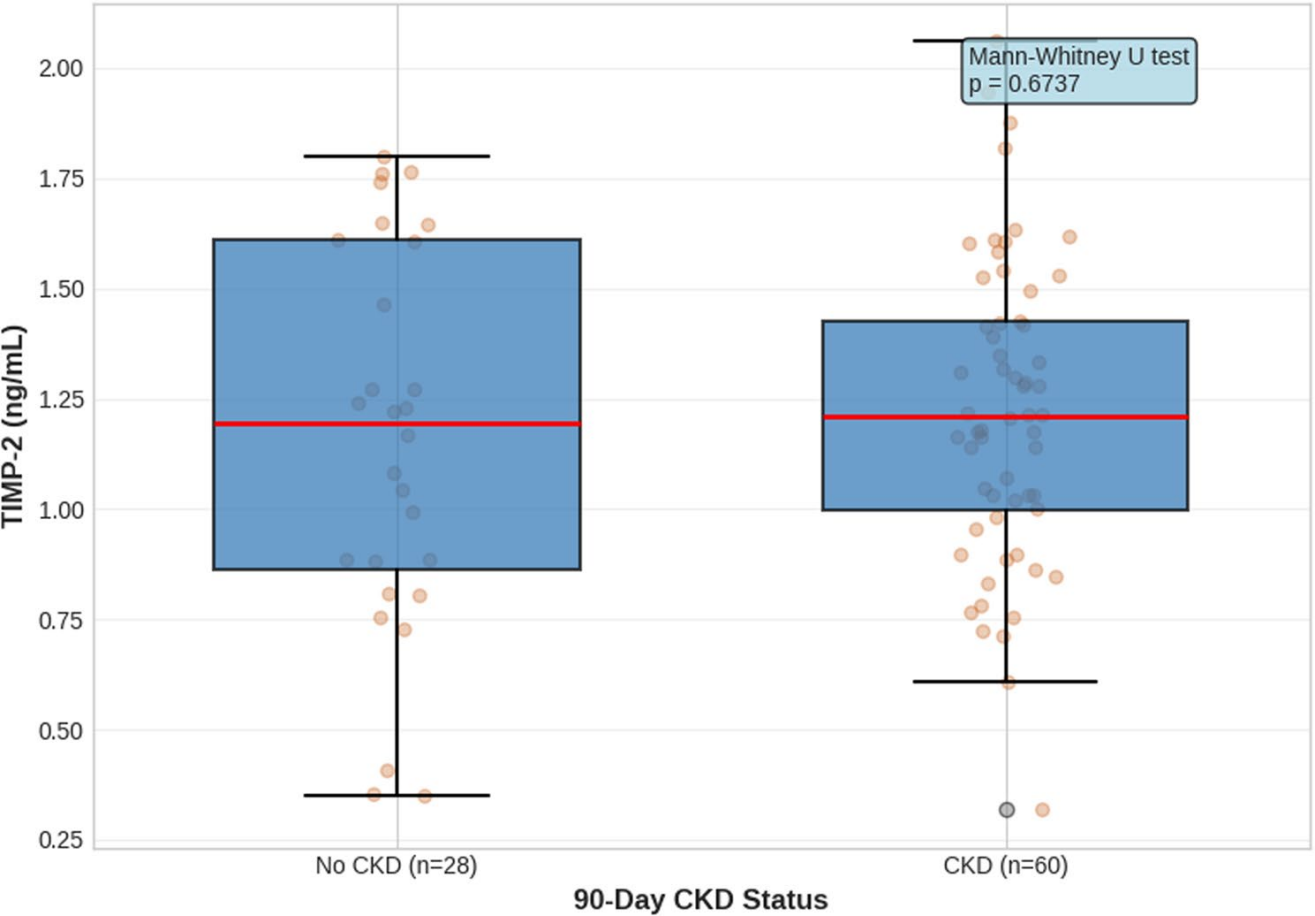
Box-and-whisker plots of 2-hour serum TIMP-2 concentrations stratified by 90-day CKD status

The distribution of 2-hour TIMP-2 levels according to 90-day CKD status is illustrated in Fig. 2.

### Correlations of TIMP-2 with renal and inflammatory markers

**Fig. 3:**
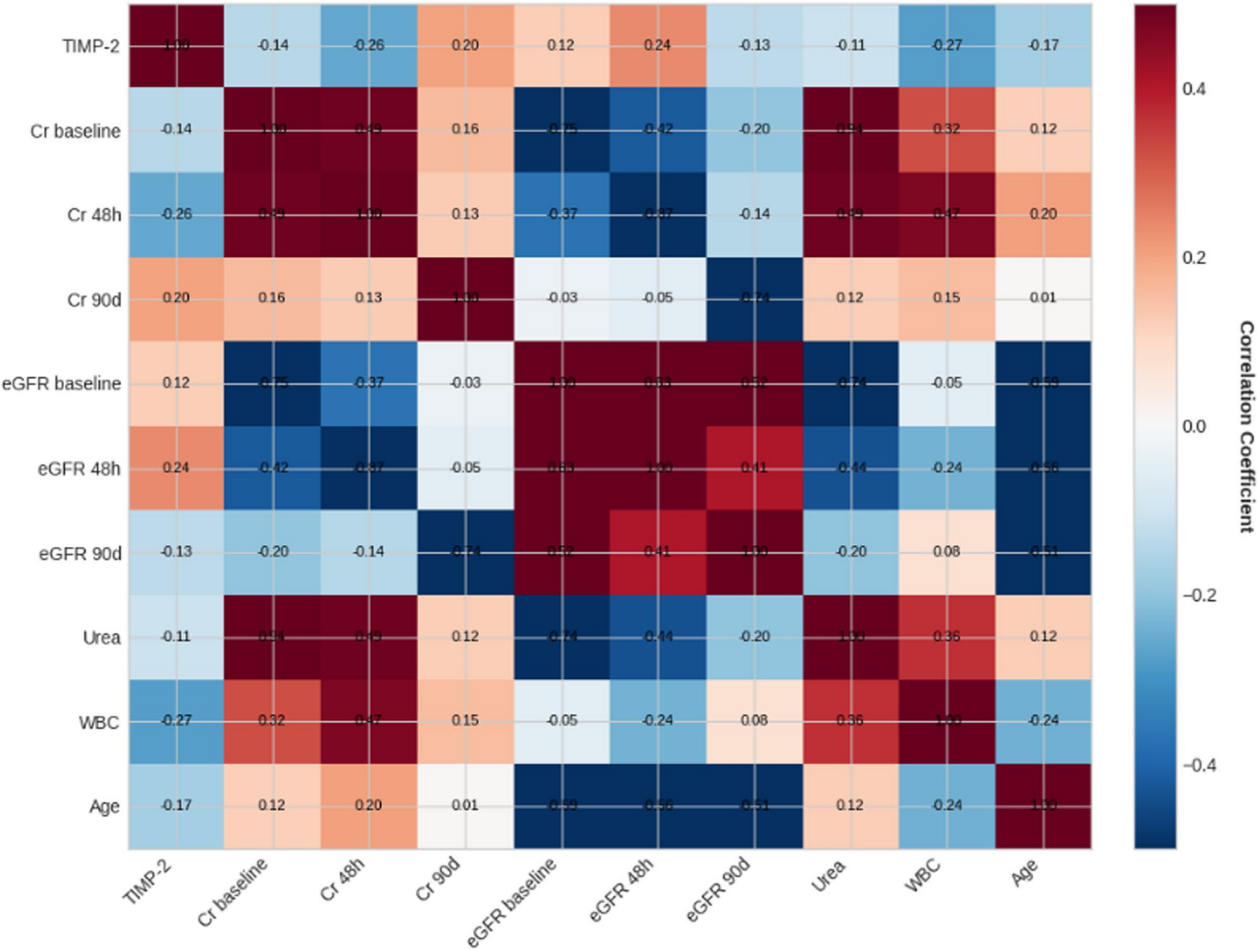
Correlation heatmap of 2-hour serum TIMP-2 with selected renal (creatinine, eGFR, urea) and inflammatory (WBC) parameters

Correlational analyses demonstrated small but directionally consistent associations between 2-hour TIMP-2 and renal function indices. TIMP-2 correlated positively with serum creatinine (*r* = 0.22; *p* = 0.04) and with urea (*r* = 0.19; *p* = 0.08), indicating that higher early tubular stress tends to co-occur with higher levels of functional renal impairment, even when values remain within or near the normal range. Correlations with inflammatory markers such as WBC count were weaker (*r* = 0.15; *p* = 0.16) and did not reach statistical significance, but the positive direction is compatible with the linkage between inflammation, tubular stress, and extracellular matrix remodelling. A correlation matrix of 2-hour TIMP-2 with key renal and inflammatory markers is presented in Fig. 3.

These modest correlation coefficients emphasise that TIMP-2 is not simply a surrogate for baseline creatinine or general systemic inflammation, but rather captures a partially distinct dimension of kidney stress that may have implications for longer-term outcomes.

### Multivariable regression analyses

In multivariable linear regression models with 90-day serum creatinine as the dependent variable, 2-hour TIMP-2 emerged as an independent predictor after adjustment for baseline creatinine and relevant clinical covariates (Table 5). Specifically, each 1-ng/mL increase in TIMP-2 was associated with a + 12.0 µmol/L increase in 90-day creatinine (B = + 12.0; *p* = 0.016), while baseline creatinine also remained significantly associated with 90-day creatinine (B = + 0.276; *p* = 0.048). These findings suggest that early tubular stress, as indexed by TIMP-2, contributes additional information beyond traditional functional markers of kidney function.

**Table 5:** Linear regression model for 90-day serum creatinine

Predictor	Regression coefficient (B)	95% CI	*p*-value
2-hour TIMP-2 (ng/mL)	+ 11.94	2.39 to 21.50	0.015
Baseline creatinine (µmol/L)	+ 0.24	-0.02 to 0.50	0.075
Age (years)	+ 0.29	-0.07 to 0.65	0.113
Sex (female)*	-14.02	-22.13 to -5.91	0.001
Contrast volume (mL)	-0.05	-0.11 to 0.01	0.089

**Fig. 4:**
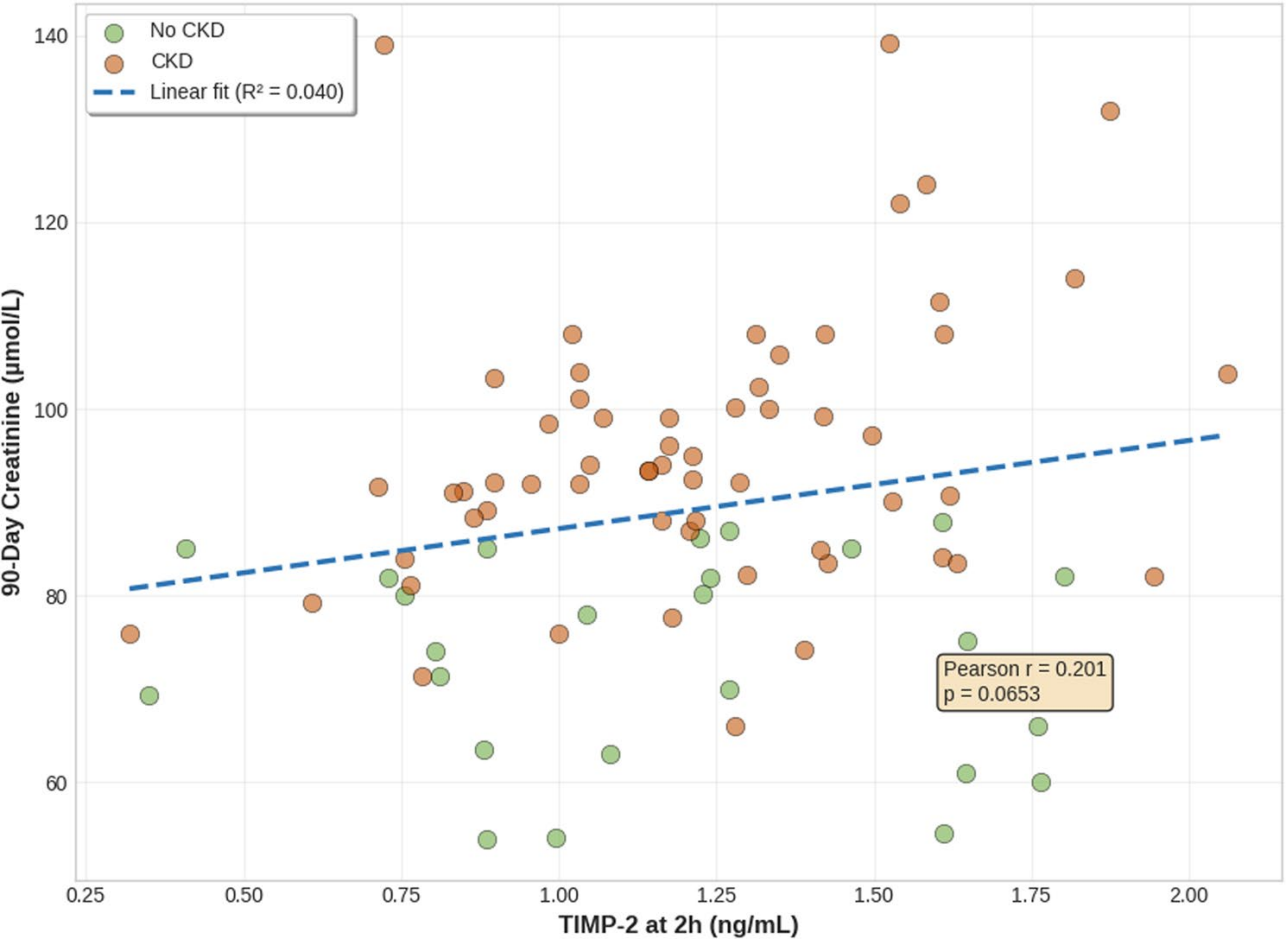
Scatter plot showing the association between 2-hour serum TIMP-2 and 90-day serum creatinine, with fitted regression line

In parsimonious logistic regression models for the binary outcome of 90-day CKD, TIMP-2 demonstrated a borderline statistical association (OR ≈ 6.38; *p* = 0.056), indicating that patients with higher TIMP-2 values were more likely to develop CKD at 90 days, albeit with limited precision due to sample size (Table 6). The relationship between 2-hour TIMP-2 and 90-day serum creatinine is shown in Fig. 4.

**Table 6:** Logistic regression model for 90-day CKD

Predictor	Odds ratio (OR)	95% CI	*p*-value
Age (per 1 year)	1.06	1.00 to 1.13	0.059
2-hour TIMP-2 (ng/mL)	4.38	0.78 to 24.50	0.093
Baseline creatinine (µmol/L)	1.00	0.96 to 1.04	0.888
Contrast volume (mL)	0.99	0.98 to 1.00	0.094

Older age was a significant independent predictor of 90-day CKD (OR 1.07 per year; *p* = 0.042), reflecting the established relationship between age, renal vulnerability, and adverse outcomes. Taken together, the regression analyses support the notion that early TIMP-2 captures clinically relevant tubular stress that influences longer-term renal trajectories, while also underscoring the ongoing importance of baseline renal reserve and demographic risk factors.

### Discrimination of TIMP-2 for 90-day CKD

**Fig. 5:**
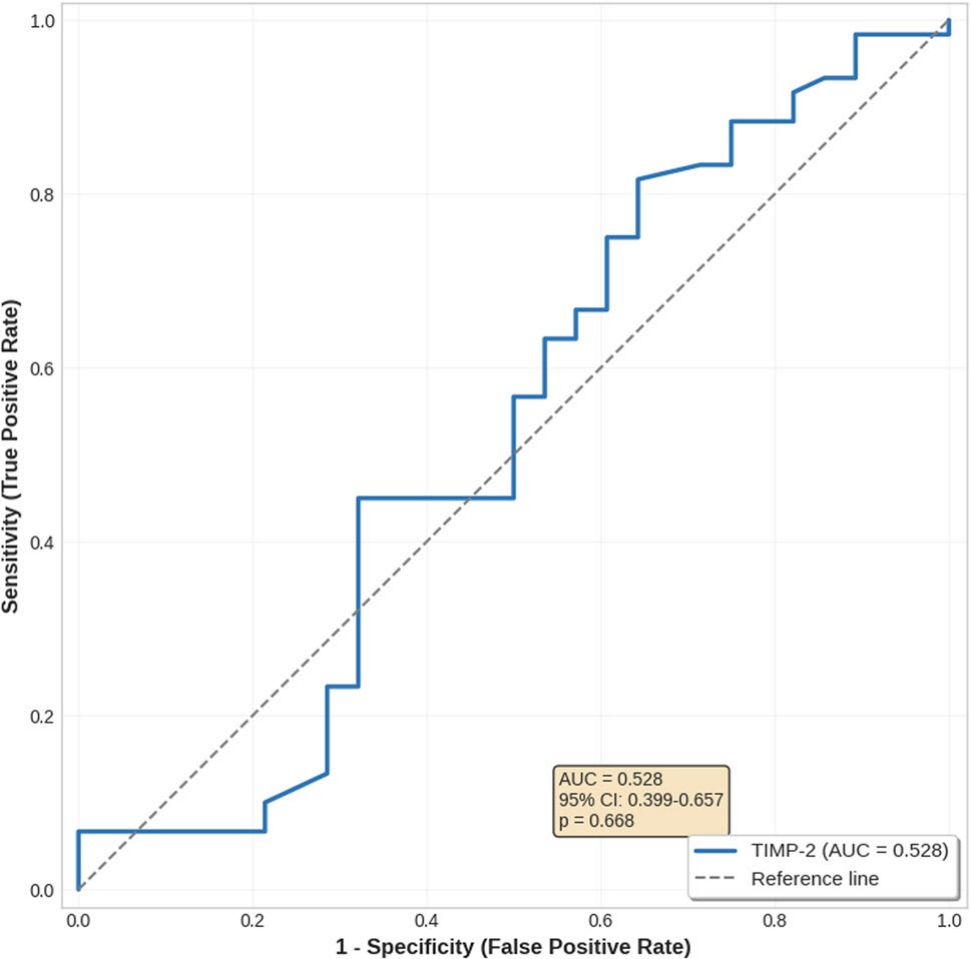
Receiver operating characteristic (ROC) curve of 2-hour serum TIMP-2 for prediction of 90-day CKD

The standalone discriminative performance of 2-hour TIMP-2 for predicting 90-day CKD was modest. ROC analysis yielded an AUC of 0.528 (95% CI 0.399–0.657; *p* = 0.668) for TIMP-2 as a univariable predictor (Table 7, Fig. 2). This AUC suggests that, on its own, early TIMP-2 only slightly outperforms random classification in distinguishing patients who will or will not develop 90-day CKD. The wide confidence interval, spanning values compatible with no discrimination, reflects the limited number of events and constrains definitive conclusions about precise performance estimates. The ROC curve of 2-hour TIMP-2 for prediction of 90-day CKD is shown in Fig. 5.

**Table 7:** Discrimination of 2-hour TIMP-2 for 90-day CKD (ROC analysis)

Predictor → Outcome	AUC	SE	95% CI	*p*-value
2-hour TIMP-2 → 90-day CKD	0.528	0.080	0.399–0.657	0.668

Overall, these results indicate that while early TIMP-2 is independently associated with 90-day creatinine and contributes to risk modelling, it is unlikely to serve as a reliable standalone classifier for long-term CKD in the emergent STEMI PCI setting. Rather, its clinical utility is more plausibly realised as part of a broader, multivariable or biomarker panel-based strategy for cardio–renal risk stratification.

## Discussion

### Principal findings

In this prospective single-centre cohort of STEMI patients undergoing emergent coronary angiography and PCI, we observed three main findings. First, the incidence of 90-day CKD (17%) substantially exceeded that of early creatinine-defined CI-AKI within 48 h (6.8%), highlighting a considerable burden of delayed renal dysfunction that is not captured by short-term monitoring alone. Second, a single 2-hour serum TIMP-2 measurement, obtained in the immediate post-procedural window, was independently associated with higher 90-day serum creatinine after adjustment for baseline creatinine and clinical covariates, indicating that early tubular stress has implications for longer-term renal trajectories. Third, despite this independent association, the standalone discriminative performance of TIMP-2 for 90-day CKD was modest (AUC ≈ 0.60), suggesting that TIMP-2 alone is insufficient as a robust classifier but may have value within multivariable or biomarker panel-based risk pathways.

### Contrast-induced AKI versus longer-term CKD in STEMI PCI

Our finding that 90-day CKD was more than twice as frequent as early CI-AKI underscores a growing recognition that traditional CI-AKI definitions, based on short-term creatinine changes, provide an incomplete picture of the overall renal impact of contrast exposure and acute myocardial infarction [37, 38]. Patients may fail to meet conventional CI-AKI thresholds yet still experience a clinically meaningful decline in renal function over the subsequent weeks to months. This is particularly relevant in STEMI PCI, where haemodynamic instability, neurohormonal activation, systemic inflammation, and therapeutic interventions (e.g., high-dose diuretics, renin- angiotensin - aldosterone system blockade) can all influence kidney function beyond the early post-contrast window [39, 40].

Our results are consistent with reports from other cardiovascular and critical care settings demonstrating that creatinine-based CI-AKI definitions may over- or underestimate true contrast-related kidney injury and that attention to longer-term renal outcomes is necessary. They support calls for more systematic assessment of eGFR and proteinuria at follow-up in high-risk cardio–renal populations, rather than focusing exclusively on early CI-AKI rates as a surrogate for renal safety.

### TIMP-2 as a marker of tubular stress: relationship to prior work

TIMP-2 is an endogenous inhibitor of matrix metalloproteinases (MMPs) and plays a key role in extracellular matrix turnover and cell-cycle regulation in response to injury. Dysregulation of the MMP/TIMP axis has been implicated in ischemia–reperfusion injury, inflammation, and interstitial fibrosis in the kidney. Cell-cycle arrest biomarker panels combining TIMP-2 and IGFBP7 ([TIMP-2]×[IGFBP7]) form the basis of the NephroCheck test and have been evaluated for early detection of AKI risk in ICU, perioperative, and emergency settings.

However, results across different populations have been heterogeneous. Some studies have reported strong short-term predictive value for moderate-to-severe AKI in critically ill patients, whereas others, including angiography and critical care cohorts, have found only limited incremental utility of cell-cycle arrest biomarkers when added to clinically based risk scores. Contextual factors such as baseline CKD, haemodynamic instability, sepsis, and timing of sampling appear to modulate biomarker performance, suggesting that no single marker can be expected to perform uniformly across diverse clinical scenarios.

Our study extends this literature in several ways. First, we focus on serum TIMP-2 alone, rather than urinary panels, in a well-defined STEMI PCI population. Serum-based assays may offer practical advantages in catheterisation laboratory workflows but have been less extensively studied. Second, we explicitly link early TIMP-2 levels to a 90-day CKD endpoint, moving beyond the prevalent focus on 24–48-h CI-AKI. The observed independent association between higher early TIMP-2 and higher 90-day creatinine supports the concept that even modest levels of tubular stress at the time of reperfusion and contrast exposure can influence the longer-term renal trajectory.

At the same time, the modest AUC of ≈ 0.60 for TIMP-2 as a univariable predictor of 90-day CKD underscores the limitations of relying on a single biomarker. In this respect, our findings align with emerging views that cell-cycle arrest markers are best utilised not as stand-alone “rule-in” or “rule-out” tests, but as components of integrated algorithms that combine clinical risk factors, established functional markers, and other biomarkers.

### Biological plausibility and interpretation of modest effect sizes

The relatively small correlation coefficients and modest discriminative performance observed for TIMP-2 are not unexpected when considering the multifactorial nature of cardio–renal interactions in STEMI. Tubular stress in this setting likely arises from multiple converging insults, including ischemia–reperfusion, neurohormonal activation, haemodynamic fluctuations, inflammatory mediators, and drug effects. TIMP-2 captures one important axis—matrix remodelling and cell-cycle arrest—but cannot fully account for all determinants of renal outcome.

The independent association of TIMP-2 with 90-day creatinine after adjusting for baseline creatinine is nonetheless clinically and biologically meaningful. It suggests that early tubular stress carries prognostic information beyond pre-existing renal function and that some patients with apparently “preserved” baseline creatinine may already be on a trajectory towards progressive renal impairment. However, given the modest effect sizes and limited sample, these observations should be interpreted as hypothesis-generating and require confirmation in larger cohorts.

### Clinical implications

From a clinical perspective, our findings support several practical points. First, in STEMI PCI patients, clinicians should be cautious about relying solely on 48-h creatinine changes to assess renal safety; a subset of patients will develop new or progressive CKD by 90 days despite not meeting CI-AKI criteria. Routine or targeted 90-day follow-up of eGFR, particularly in older patients and those with reduced baseline eGFR or high contrast exposure, may allow earlier identification and management of emerging CKD.

Second, a single early serum TIMP-2 measurement may aid in refining risk assessment by identifying patients with subclinical tubular stress who are at increased risk of adverse renal trajectories. However, based on our data, TIMP-2 should not be used in isolation as a binary decision tool for predicting 90-day CKD; its modest AUC indicates that many patients would be misclassified if TIMP-2 were applied as a sole screening test.

Third, the greatest potential value of TIMP-2 likely lies in its integration into multivariable risk models or biomarker panels. Combining TIMP-2 with clinical covariates (age, sex, Killip class), baseline eGFR, contrast volume, and perhaps other tubular or inflammatory markers could yield more accurate prediction tools that better reflect the complex pathophysiology of cardio–renal injury in STEMI.

### Strengths and limitations

Strengths of our study include the prospective design, enrolment of a consecutive real-world STEMI PCI population, and standardized timing and pre-analytic handling of TIMP-2 samples. We used widely available, validated laboratory methods for both routine parameters and TIMP-2 quantification. Importantly, we focused on a 90-day CKD endpoint, which is rarely reported in contrast nephropathy research dominated by short-term CI-AKI outcomes, thus providing a more comprehensive view of renal sequelae after emergent PCI.

Several limitations must be acknowledged. This was a single-centre study with a relatively small sample size and a limited number of 90-day CKD events, which restricts the precision of our estimates and limits the complexity of multivariable models to avoid overfitting. TIMP-2 was measured at a single 2-hour timepoint; serial measurements might better capture the temporal dynamics of tubular stress and identify optimal sampling windows for prediction. We did not measure other biomarkers such as NGAL or IGFBP7 in parallel, precluding direct comparison between single-marker and panel approaches. Follow-up creatinine and eGFR at 90 days were obtained through a combination of scheduled visits and external laboratory results, which may introduce variability in measurement timing and laboratory methods. Residual confounding by unmeasured factors, including medications, intercurrent illnesses, or unrecorded episodes of hypotension, cannot be excluded.

### Future directions

Future research should build on these findings in several directions. Larger, multicentre cohorts of STEMI and high-risk PCI patients with systematic 90-day (and longer) renal follow-up are needed to validate the observed association between early serum TIMP-2 and long-term kidney function and to refine estimates of predictive performance. Studies incorporating serial biomarker sampling at multiple timepoints (e.g., pre-contrast, 2 h, 6–12 h, 24–48 h) could elucidate the temporal profile of tubular stress and identify optimal windows for risk stratification.

Combining TIMP-2 with complementary biomarkers (such as IGFBP7, NGAL, or markers of inflammation and endothelial dysfunction) in penalised regression or machine-learning models, with appropriate internal and external validation, may yield more powerful and clinically useful prediction tools. Interventional studies may ultimately be required to determine whether biomarker-guided strategies—such as intensified nephroprotection, closer monitoring, or tailored pharmacotherapy—can improve hard renal and cardiovascular outcomes in high-risk STEMI PCI populations.

## Conclusions

In this prospective cohort of STEMI patients undergoing emergent coronary angiography and PCI, 90-day CKD occurred nearly three times more often than early creatinine-defined CI-AKI, revealing a substantial burden of delayed renal dysfunction that is not captured by short-term monitoring alone. A single 2-hour serum TIMP-2 measurement, obtained immediately after the index procedure, reflected early tubular stress and was independently associated with higher 90-day creatinine beyond baseline renal function and clinical covariates.

However, the standalone discriminative performance of TIMP-2 for predicting 90-day CKD was modest, indicating that TIMP-2 alone is unlikely to serve as a reliable binary classifier in clinical practice. These findings support the implementation of extended renal follow-up in high-risk STEMI PCI populations and suggest that TIMP-2 is best considered as a component of multivariable or biomarker panel-based risk stratification pathways, rather than as an isolated test. Larger multicentre studies with serial biomarker assessment are warranted to define the optimal role of TIMP-2 within integrated cardio–renal risk algorithms.

## Data Availability

The data generated in this study are available upon request from the corresponding author.
